# The current state of research for psychobiotics use in the management of psychiatric disorders–A systematic literature review

**DOI:** 10.3389/fpsyt.2023.1074736

**Published:** 2023-02-23

**Authors:** Octavian Vasiliu

**Affiliations:** Department of Psychiatry, Dr. Carol Davila University Emergency Central Military Hospital, Bucharest, Romania

**Keywords:** probiotics, prebiotics, synbiotics, schizophrenia, major depressive disorder, neurocognitive disorders, substance use disorders, autism spectrum disorders

## Abstract

The need to find new therapeutic interventions in patients diagnosed with psychiatric disorders is supported by the data suggesting high rates of relapse, chronic evolution, therapeutic resistance, or lack of adherence and disability. The use of pre-, pro-, or synbiotics as add-ons in the therapeutic management of psychiatric disorders has been explored as a new way to augment the efficacy of psychotropics and to improve the chances for these patients to reach response or remission. This systematic literature review focused on the efficacy and tolerability of psychobiotics in the main categories of psychiatric disorders and it has been conducted through the most important electronic databases and clinical trial registers, using the PRISMA 2020 guidelines. The quality of primary and secondary reports was assessed using the criteria identified by the Academy of Nutrition and Diabetics. Forty-three sources, mostly of moderate and high quality, were reviewed in detail, and data regarding the efficacy and tolerability of psychobiotics was assessed. Studies exploring the effects of psychobiotics in mood disorders, anxiety disorders, schizophrenia spectrum disorders, substance use disorders, eating disorders, attention deficit hyperactivity disorder (ADHD), neurocognitive disorders, and autism spectrum disorders (ASD) were included. The overall tolerability of the interventions assessed was good, but the evidence to support their efficacy in specific psychiatric disorders was mixed. There have been identified data in favor of probiotics for patients with mood disorders, ADHD, and ASD, and also for the association of probiotics and selenium or synbiotics in patients with neurocognitive disorders. In several domains, the research is still in an early phase of development, e.g., in substance use disorders (only three preclinical studies being found) or eating disorders (one review was identified). Although no well-defined clinical recommendation could yet be formulated for a specific product in patients with psychiatric disorders, there is encouraging evidence to support further research, especially if focused on the identification of specific sub-populations that may benefit from this intervention. Several limitations regarding the research in this field should be addressed, i.e., the majority of the finalized trials are of short duration, there is an inherent heterogeneity of the psychiatric disorders, and the diversity of the explored Philae prevents the generalizability of the results from clinical studies.

## 1. Introduction

The communication between the gut microbiome (GM) and the central nervous system (CNS) involves multiple neuro-immune and metabolic circuits *via* the vagal pathway or through the GM-synthesized metabolites, gut hormones, and endocrine peptides (1). Therefore, maintaining a healthy GM is currently explored as an essential factor for preserving mental health. The administration of prebiotics, synbiotics, or probiotics has been researched in patients with vulnerability toward-, or well-established diagnoses of psychiatric disorders and also in preclinical models of these conditions (1).

The diversity of GM and taxa abundance changes have been explored in clinical settings, and the results support the existence of a difference between patients (e.g., those diagnosed with depressive disorders, psychotic disorders, substance use disorders, etc.) and the general population (2–4). The association between GM changes and the onset or persistence of psychiatric disorders is difficult to explain because most of the discoveries related to GM composition are made after the onset of a specific pathology. To make things even more complicated, several psychotropics have been associated with changes in GM diversity, e.g., antipsychotics may exert a dose-related negative effect on the Shannon index and phylogenic diversity (5). Also, antidepressants exert *in vitro* changes in the representation of various GM species, most of their effects being antimicrobial (6).

High relapse rates, various types of disability, increased non-adherence, and treatment resistance have been reported across the spectrum of psychiatric disorders (7, 8). These negative prognosis factors indicate the need to find new therapeutic interventions for patients diagnosed with psychiatric disorders and even for the prophylaxis of such disorders. In order to validate the current state of knowledge regarding the efficacy and adverse events profile of psychobiotics in the treatment and/or prevention of psychiatric disorders, a review of the literature was conducted. Within this review, the category of “psychobiotics” includes probiotics (bacteria), prebiotics (non-digestible oligosaccharides), and synbiotics (various combinations of the previous products) (9). The definition of psychobiotics, according to Dinan et al. (10), is “a live organism that, when ingested in adequate amounts, produces a health benefit in patients suffering from psychiatric illness.” Adding the dimension of GM modulation, Del Toro-Barbosa et al. (11) consider that “psychobiotics are defined as probiotics that confer mental health benefits to the host when ingested in a particular quantity through interaction with commensal gut bacteria.” Also, the good tolerability of psychobiotics makes them more useful for a population with well-known adverse events to their ongoing medication (e.g., weight gain, diabetes, dyslipidemia, extrapyramidal manifestations, etc.) (11, 12). It is expected that psychobiotics would be a viable add-on option for patients diagnosed with psychiatric disorders due to their low risk of secondary effects, allergies, or dependence (11). *Probiotics* are considered “viable microorganisms, sufficient amounts of which reach the intestine in an active state and thus exert positive health effects” (9). There are many strains used in probiotic food, especially fermented milk products, e.g., lactobacilli, bifidobacteria, enterococci, streptococci, strains of Escherichia coli, etc. *Prebiotics* are “selectively fermented ingredients that allow specific changes both in the composition and/or activity in the gastrointestinal microflora that confer benefits upon host wellbeing and health” (9). From this category of psychobiotics, bifidogenic, non-digestible oligosaccharides are the most extensively explored products (9). *Synbiotics* are synergistic combinations of pro- and prebiotics (9).

The exact mechanisms by which psychobiotics exert their action are incompletely described, but induction of immunomodulatory mechanisms, protective effects against physiological stress, inhibition of pathogens growth, microbiome modulation, and improvement of the barrier function of the colonic epithelium have been explored (13).

A series of challenges have been reported by researchers investigating the effects of psychobiotics in clinical practice. The high heterogeneity of the microorganisms investigated and products administered during various clinical and preclinical studies, the paucity of well-designed clinical trials, especially of long duration, as well as the need to better define target subpopulations are but a few of the challenges faced by the research of psychobiotics (13, 14).

## 2. Objectives

The main objective was to evaluate the efficacy and tolerability of pre, pro, and synbiotics in different psychiatric disorders, based primarily on data derived from quantitatively and mixed (quantitatively and qualitatively) research.

A secondary objective was defined as the possibility of formulating a clinical recommendation for the use of psychobiotics in patients with psychiatric disorders in accordance with evidence found for their efficacy and tolerability.

## 3. Methodology

Due to the relative novelty of the subject, the methodology was conceived to include the largest pool of data available, meaning preclinical and clinical research derived from both primary and secondary reports (i.e., different types of studies or clinical cases and various types of reviews).

### 3.1. Design and search strategy

A systematic review focused on the efficacy and adverse effects of pre, pro, and synbiotics in the case of psychiatric disorders was conducted, based on PRISMA 2020 guidelines (15). The main electronic databases (PubMed, Cochrane, EMBASE, Clarivate/Web of Science) were included. Also, the register of clinical trials run by the US National Library of Medicine (NLM)^1^ was searched for potential data regarding finalized studies dedicated to this subject.

The search paradigm used was “prebiotics” OR “probiotics” OR “synbiotics” OR “psychobiotics” AND “mood disorders” OR “major depression” OR “bipolar disorder” OR “schizophrenia” OR “substance use disorders” OR “anxiety disorders” OR “eating disorders” OR “neurocognitive disorders” OR “autism” OR “ADHD” OR “psychiatric disorders.” All papers published between January 1990 and July 2022 were included in the primary search.

The checklist for the PRISMA criteria has been presented in Supplementary Table 1.

### 3.2. Inclusion and exclusion criteria

All reports referring to clinical or cohort studies, case reports, reviews, meta-analytic investigations, and preclinical research were allowed. Interventions assessed were probiotics, prebiotics, and/or synbiotics, without limitations regarding their composition or duration of administration. Patients diagnosed with any psychiatric disorder were allowed as participants if the diagnoses were based on specified criteria. Also, for preclinical studies, the model of a psychiatric disorder should be specified. The outcomes were assessed on psychometric validated scales or clinical observation for clinical trials and secondary reports and on specific behavioral manifestations for preclinical studies. Studies reporting gut microbiome changes, anthropometric markers, and/or biological variables (e.g., pro-inflammatory markers, brain-derived neurotrophic factor- BDNF, etc.) were also reviewed. Only reports written in English, for which the full paper could be accessed were included.

Exclusion criteria refer to studies without a clearly specified methodology (e.g., duration, methods of assessment, inclusion/exclusion criteria), participants without a psychiatric disorder or without a well-defined behavioral model for a psychiatric disorder in the case of preclinical studies, interventions other than those previously mentioned (e.g., fecal microbiota transplant), reports written in other languages than English, and purely qualitative research (e.g., expert opinion, perspectives, conceptual analyses).

### 3.3. The assessment of evidence quality

The quality of evidence was based on criteria identified by the Academy of Nutrition and Diabetics for primary and secondary reports (16). These criteria are derived from the Agency for Healthcare Research and Quality guideline on rating systems for the strength of scientific evidence (17). The checklist for each research includes four relevance questions and 10 validity questions (17, 18). This methodology was preferred because it refers to both human trials and animal studies, and it includes criteria for the quality evaluation of observational, interventional, prospective, and retrospective studies, case reports, meta-analyses, and reviews. The quality of each research is scored “positive” (no risk of bias identified, very good methodology), “neutral” (the research is neither very accurate nor extremely weak), or “negative” (the main methodological aspects have not been adequately assessed), based on the quality criteria checklist (16). The reports are classified as “A” (randomized controlled/crossover trials), “B” (prospective/retrospective cohort study), “C” (non-randomized controlled/crossover trials, case-control studies), “D” (non-controlled studies, case studies, other descriptive research), “M” (meta-analyses, systematic reviews), “R” (narrative reviews, consensus statements) or “X” (medical opinions) (16).

## 4. Results

The primary search identified 1,062 reports, but only 43 remained after filtering them out according to the inclusion and exclusion criteria (Supplementary Figure 1). When distributed to different categories of psychiatric disorders, a degree of overlap between studies was detected because several reports included outcomes referring to multiple psychiatric manifestations (Table 1). Reports about the effects of psychobiotics on mood disorders were identified in 12 sources, while data about the modulation of anxiety manifestations through this type of intervention was found in nine sources (partially overlapping). References about schizophrenia spectrum disorders (SSD), substance use disorders (SUDs), neurocognitive pathology, and eating disorders were included in five, three, seven, and one reports, respectively. The impact of psychobiotics in patients with autism spectrum disorders (ASDs) or ADHD was also assessed in six and three reports, respectively.

**TABLE 1:** Identified reports on the efficacy and tolerability of psychobiotics in patients with psychiatric disorders and their overall quality of evidence.

References	Study type	Population	Intervention	Outcomes	Duration of treatment	Results	Observations	Class	OQR
**Mood disorders**									
(20)	A systematic review of human studies (*n* = 13 trials)	Adults with MDD/BD	Probiotics *(Bifid.* and *Lact.* spp.)	Depressive symptoms	4-24 weeks	Seven trials concluded in favor of the intervention, and six did not.	Three positive results studies on *Lact. gasseri* were conducted by the same group of researchers. Not all probiotic bacteria could be efficient in decreasing depression severity.	M	ø
(21)	Systematic review (*n* = 3 trials) and meta-analysis (*n* = 2 RCTs)	713 women in the systematic review and 545 in the meta-analysis, all were pregnant at baseline	Probiotics *(Lact.* and/or *Bifid.* spp.) vs. placebo	EPDS scores	4-24 weeks	No significant difference was recorded in the active vs. placebo groups regarding the main outcome, or in the global mental health scores.	Anxiety levels were reduced more by the probiotics vs. placebo.	M	+
(23)	DBRCT	423 women, 14-16 weeks of gestation at baseline	Probiotics *(Lact. rhamnosus)* vs. placebo	EPDS and STAI-6 scores	∼24 weeks	Decreased depression/anxiety scores more in the active vs. placebo participants at the endpoint.	The number of women with clinically significant levels of anxiety was lower in the active group.	A	+
(24)	Systematic review (*n* = 62 trials) and meta-analysis (*n* = 50 RCTs)	Adults	Pre *(Lact., Bifid., Bacillus, Cl., Lactococcus, Strep., Weisella, Lacticaseibacillus)*, pro, or synbiotics vs. placebo	Depressive symptoms measured on a validated scale	Variable, but most of the trials included had a duration of < 24 weeks	The results favored the active intervention based on the main outcome.	Effect sizes for synbiotics were larger than for prebiotics or probiotics.	M	+
(25)	OLT	Adults, 40 participants with MDD	*Cl.butyricum + ADT* (SSRIs or duloxetine)	HDRS-17, BDI, and BAI scores	8 weeks	70% response rate, 35% remission rate. The overall tolerability was good. No SAE was reported.	All enrolled patients were completers.	D	ø
(26)	Cross- sectional populational study	Adult subjects (*N* = 18019)	Probiotic foods, probiotic supplements	PHQ-9 scores	Variable exposure (at least 30 days prior to one of the two study visits)	The use of probiotics was correlated with a diminished risk of depression according to the unadjusted data. After data adjustment, the prophylactic effect of the probiotics was no longer significant.	The monitoring period was 8 years (2005– 2012).	D	-
(27)	DBRCT	Adults with MDD (*N* = 110)	Probiotics *(Lact. helveticus, Bifid. longum)* or prebiotic (galactooligosaccharide) vs. placebo	BDI scores	8 weeks	No significant difference at the endpoint between groups for prebiotics. Probiotic supplementation improved significantly the primary outcome vs. placebo.	The Trp/Ile increased significantly during the prebiotic administration vs. placebo.	A	+
(28)	DBRCT	Adults with moderate and severe mood symptoms, currently not under ADT treatment (*N* = 79)	Probiotics *(Lact.* *helveticus, Bifid.* *longum)* vs. placebo	MADRS scores	8 weeks	No significant difference at the endpoint between groups.	Baseline vitamin D level moderated the treatment effect on multiple outcome measures.	A	+
(29)	DBRCT	MDD patients (*N* = 40)	Probiotics *(Lact., Bifid.)* vs. placebo	BDI scores, multiple biological variables	8 weeks	The BDI scores significantly improved vs. placebo. Insulin, HOMA-IR, and serum hs-CRP levels also decreased significantly in the active group vs. placebo	The glutathione levels increased significantly in patients receiving probiotics. No change was reported for fasting plasma glucose, insulin sensitivity check index, lipid profiles, or other metabolic parameters.	A	+
(30)	Permuted block RCT	Type 1 BD patients (*N* = 38)	Probiotics *(Bifid., Lact.)* vs. placebo	HDRS and YMRS scores	8 weeks	No significant differences at the endpoint between groups in the primary outcome, but a trend toward superiority for probiotics was reported.	Small sample size, and possible interactions between mood stabilizers and probiotics.	A	ø
(31)	Triple-blind RCT	Adults with depressive symptoms (*N* = 71)	Probiotics (a mixture of *Bifid.* spp., *Lacto.* spp., *and Lactococcus lactis*)	BDI, BAI, LEIDS-R, DASS-21 scores	8 weeks	No significant effect of probiotics on depressive or anxiety severity.	High attrition rate (34%).	A	ø
(32)	DBRCT	Patients undergoing hemodialysis (*N* = 75)	Synbiotic and probiotic *(Lact. acidophilus, Bifid.* spp.) vs. placebo	HADS-ANX, BDNF serum level HADS-DEP,	12 weeks	Synbiotics determined a significant decrease in HADS-DEP scores in patients with depressive symptoms vs. placebo and vs. probiotics. Also, synbiotics decreased HADS-DEP scores in all patients vs. placebo.	In patients with depressive symptoms, BDNF levels increased significantly in the synbiotic group vs. placebo and vs. probiotic groups.	A	+
**Anxiety disorders**									
(22)	Systematic review (*n* = 3 trials) and meta-analysis (*n* = 2 RCTs)	713 participants in the systematic review and 545 in the meta-analysis, women during pregnancy	Probiotics *(Lact., Bifid.)*	STAI-6 scores	4-24 weeks	Anxiety levels were reduced more by the probiotics vs. placebo.	Depression scores were not significantly improved by the probiotics vs. placebo	M	ø
(35)	Pilot, DBRCT	Pregnant women with severe depressive and/or anxiety manifestations (*N* = 40)	Probiotics *(Lact, Lactococcus, Bifid.)* vs. placebo	EPDS, LIDS- R, PRAQ-R, STAI, PES, EPL, MAAS, and MPAS scores	8 weeks	No significant difference was reported between groups at the endpoint regarding any of the outcome measures. The tolerability of probiotics was good.	This was a pilot trial so a low number of subjects were randomized in each arm.	A	ø
(31)	Triple-blind RCT	Adults with depressive symptoms (*N* = 71)	Probiotics (a mixture of *Bifid., Lacto., and Lactococcus lactis*)	BDI, BAI, LEIDS-R, DASS-21 scores	8 weeks	No significant effect of probiotics on anxiety severity.	High attrition rate (34%).	A	ø
(36)	A systematic review (*n* = 12 studies)	Adults with a high level of stress, anxiety, or depression	Probiotics (*Bifid., Lact., Strep. salivarius/ termophilus, Cl. butyricum, Lactococcus* spp.), prebiotics, and/or synbiotics vs. placebo	Different scales for anxiety, stress, and depression	3-8 weeks	Anxiety levels were decreased by the probiotics.	Only two studies confirmed the efficacy of probiotics in patients with anxiety. Five trials did not support any improvement in this domain.	M	ø
(37)	DBRCT	Healthy volunteers (*N* = 150)	Probiotic mixture *(Strep. thermophiles, Bifid., and Lact.* spp.) vs. placebo	HAMA	12 weeks	The HAMA score decreased significantly vs. the placebo	The status of IL-1 beta rs 16944 carrier correlated with a favorable effect during probiotics administration.	A	+
(38)	DBRCT	Patients with GAD (*N* = 48)	Probiotic mixture *(Bifid.* spp., *and Lact. acidophilus)* vs. placebo + sertraline	HAMA	8 weeks	The primary outcome measure was improved by probiotic use vs. placebo.	The quality of life was not affected by the probiotic intervention.	A	+
(32)	DBRCT	Patients undergoing hemodialysis (*N* = 75)	Synbiotic and probiotic *(Lact. acidophilus, Bifid.* spp.) vs. placebo	HADS-ANX, HADS-DEP, BDNF serum level	12 weeks	Synbiotics did not improve significantly HADS-ANX scores vs. placebo, but all patients had a favorable evolution when compared to baseline. Patients with depressive symptoms also presented a favorable evolution vs. baseline during synbiotics use.	HADS is a self-evaluated scale, and no other validated scales have been used.	A	+
(39)	DBRCT	Healthy volunteers (*N* = 60)	Probiotics (various brands, composition not reported) vs. placebo	BAI and other scales validated for anxiety measurement	4 weeks	Probiotics improved panic anxiety, neurophysiological anxiety, negative affect, and worry.	Patients with a high level of distress had a better evolution during the probiotic administration. A ceiling effect is possible in this study for the anxiety-related variables.	A	+
(40)	DBRCT	Adults with moderate stress levels (*N* = 111)	Probiotics *(Lact. plantarum)* vs. placebo	DASS-42 scores, multiple biological markers (e.g., plasma cortisol, cytokines levels, etc.)	12 weeks	The probiotics significantly decreased manifestations of stress, anxiety, and total psychological scores starting from week 8 vs. the placebo	Psychological functions, cognitive health, and memory are improved by probiotics in stressed adults.	A	+
**Schizophrenia spectrum disorders**									
(49)	Meta- analysis (*n* = 28 RCTs)	Patients with SCHZ	Psychobiotics, antibiotics, and antimicrobials vs. placebo as add-on	PANSS scores as the main outcome	12-24 weeks for the psychobiotics trials	No significant improvements during probiotic use were observed in the domain of negative symptoms. Vitamin D + probiotics may be superior to the placebo for negative symptoms management. Cognitive symptoms may be improved vs. placebo at 24 weeks. The tolerability of probiotics was similar to placebo.	Only three trials included pre/probiotics, one of which did not assess the negative symptoms.	M	ø
(50)	DBRCT	Patients with chronic SCHZ (*N* = 58)	Probiotics *(Lact.* *rhamnosus, Bifid.* *animalis)* vs. placebo as an add-on	Serum proteins related to immunity level determined in the blood, and BDNF serum level	14 weeks	Probiotics led to tvon Willebrand factor, | MCP- **l**,**t** BDNF, **t** RANTES, and **t** MIP-ip	Probiotics might exert their effects by regulating immune and intestinal epithelial cell functions via IL-17.	A	ø
(51)	DBRCT	Outpatients with SCHZ with moderate-severe symptoms (*N* = 65)	Probiotics *(Lact.* and *Bifid.* spp.) vs. placebo	PANSS scores	14 weeks	No significant difference in the PANSS total scores was detected between groups at the endpoint.	Patients treated with probiotics developed less frequently severe bowel symptoms during the trial.	A	ø
(52)	OLT	Outpatients with SCHZ (*N* = 29)	Probiotics *(Bifid. breve)*	HADS, PANSS - anxiety/ depression scores	4 weeks, FU visit at week 8	HADS and PANSS- anxiety/ depression scores decreased significantly after 4 weeks; 12 patients were responders.	Responders also presented fewer negative symptoms and a higher relative abundance of *Parabacteroides* in the GM vs. non-responders.	C	ø
(53)	DBRCT	Patients with chronic SCHZ (*N* = 60)	Vitamin D3 and probiotics vs. placebo as add-on	PANSS total and general scores, antioxidant markers, metabolic and inflammatory variables	12 weeks	PANSS scores improved after 12 weeks.	Antioxidant markers increased vs. placebo. Metabolic and inflammatory parameters improved vs. placebo.	A	+
**Substance use disorders**									
(55)	An animal model study, C57BL/6 mice	Chronic binge alcohol exposure	Synbiotic vs. placebo	GM composition, hepatocyte lesions	10 days	In female mice who received chronic-binge ethanol feeding for ten days, the GM decreased its abundance and diversity, and the hepatocytes were more damaged than in mice receiving gavage with saline solution.	The synbiotics administered in mice exposed to alcohol use reduced the impact of this drug on the GM and liver endothelial barrier integrity.	A	ø
(56)	An animal model study, C57BL/6 mice	Chronic binge alcohol exposure	Synbiotic *(Faecalibacterium* *prausnitzii*+ potato starch) vs. fecal slurry	Hepatic inflammatory markers and oxidative stress variables	10 days	A decreased hepatic steatosis was induced by alcohol exposure when synbiotics were concomitantly administered.		A	ø
(57)	An animal model study, Wistar rats		A normal liquid diet +/- synbiotic or an ethanol liquid diet +/- synbiotic supplementation	Hepatic inflammatory markers and oxidative stress variables	12 weeks	The addition of a synbiotic attenuated the plasma endotoxin, hepatic triglyceride, and TNF-α levels, and increased the hepatic IL-10 concentration.	The synbiotic also protected against alcohol-determined increased permeability of the intestine and higher concentration of *Bifid.* and *Lacto.* in the feces.	A	ø
**Neurocognitive disorders**									
(62)	Meta-analysis (*n* = 3 RCTs)	Patients with AD (*N* = 161)	Probiotics *(Lacto. and Bifid.* spp.) and synbiotics	Psychometric measurements and metabolic variables	12 weeks	No significant cognitive improvement was reported during the administration of the probiotic.	The quality of evidence was very low for the cognitive outcome.	M	+
(63)	OLT	Patients with AD (*N* = 16)	Probiotics (fermented milk supplement containing *Acetobacter* spp., *Lact.* spp., *Enterococcus faecium, Leuconostoc* spp., *Candida* spp.)	MMSE and other seven validated instruments for the assessment of cognitive functioning, cytokines serum levels, and oxidative processes markers.	90 days	Improvements in memory, visual-spatial/abstraction abilities, and executive/language functioning were observed at the end-point, and the level of several inflammatory cytokines and oxidative stress markers decreased.	No control group using other probiotics, and a very small sample size	C	ø
(64)	DBRCT	Healthy elders (*N* = 49)	Synbiotics (fructooligosaccharide + *Lact.* spp., *Bifid. lactis*) vs. placebo	GDS-15, MMSE, and inflammatory and oxidative stress markers	24 weeks	The effects of synbiotics on depressive symptoms and cognitive functioning were modest at six months vs. placebo.	GDS-15 scores reflected a slight worsening of depressive symptoms in both groups and a slight improvement in the MMSE scores.	A	+
(65)	DBRCT	Patients with AD (*N* = 79)	Probiotics (*Lact.+ selenium, Bifid.* spp.)+ selenium vs. selenium vs. placebo	Inflammatory and metabolic markers	12 weeks	Probiotic+selenium led to a significant reduction of hs-CRP and an increase in total antioxidant capacity and total glutathione vs. selenium as monotherapy or placebo. Serum levels of triglycerides, VLDL, LDL, and total-/HDL-cholesterol, were significantly reduced by this combination of selenium and probiotics vs. selenium as monotherapy and placebo.		A	+
(66)	DBRCT	Older adults with MCI (*N* = 80)	Probiotics *(Bifid. breve)* vs. placebo	RBANS and JMCIS scores	16 weeks	The cognitive functioning improved significantly in individuals using probiotics vs. placebo at the endpoint. Immediate memory, visuospatial/constructional, and delayed memory were significantly improved, as well as the global cognitive score.		A	+
(67)	DBRCT	Healthy participants (*N* = 63)	Probiotics *(Bifid.* spp.)	Cognition and mood symptoms, GM composition, BDNF serum level	12 weeks	The relative abundance of GM species with pro-inflammatory roles decreased significantly during probiotic treatment. Mental flexibility and stress scores were also improved by probiotics vs. placebo. BDNF levels increased also in the active intervention group.		A	+
(68)	DBRCT	Elderly individuals with cognitive complaints (*N* = 121)	Probiotics *(Bifid. breve)* vs. placebo	RBANS and MMSE scores	12 weeks	A significant improvement was recorded in both groups, without differences between interventions. Immediate memory was, however, more improved under probiotics vs. placebo, both according to the RBANS and MMSE tests, but only in subjects with low RBANS scores at baseline. The tolerability of probiotic supplementation was good.		A	+
**Eating disorders**									
(73)	Review (*n* = 28 RCTs)	Patients with obesity	Pre, pro, and synbiotics vs. placebo	Metabolic and anthropometric parameters	6-28 weeks	Prebiotic use had a neutral effect on BW, with the possible reduction of inflammatory markers. Probiotics had a significant minor impact on BW and metabolic parameters.	Changes in GM were reported irregularly with pre or probiotics.	R	+
**Autism spectrum disorders**									
(78)	Systematic review (*n* = 14 controlled and uncontrolled clinical trials)	Children with ASD (sample sizes from 10 to 85)	Pro and/or prebiotics, or FMT	Behavioral outcomes measured on specific, validated scales	3 weeks-6 months	Probiotics did not influence positively the GM on RCTs. Prebiotics and synbiotics may be efficacious in improving specific behavioral symptoms, based on data from non-randomized controlled trials.	Only five RCTs had high methodological quality.	M	+
(76)	Narrative review (*n* = 5 controlled and uncontrolled trials)	Children with ASD (*N* = 117)	Probiotics *(Lact.* spp., *Bifid.* spp., *Strep.)*, mostly blended formulations compared or no with placebo	Behavioral and general symptoms using validated scales and clinical reports, GM composition	3 week-4 months	Probiotics may be helpful for ASD patients, and they may alter the GM or urine metabolites in a beneficial direction while reducing the ASD symptoms severity.	The available data are of poor methodological quality and allow for multiple confounding factors.	R	ø
(79)	DBRCT	ASD children (*N* = 22)	Probiotics *(Lacto.* *plantarum)* vs. placebo	DBC scores, GM composition, diary with clinical symptoms	3 weeks, with a monitoring period of 12 weeks	Probiotics were associated with significant changes in DBC scores. Probiotics also led to a substantial increase of lactobacilli and enterococci groups while significantly decreasing *Cl. cluster XIVa* vs. placebo.	A very high rate of dropouts was reported, and a higher inter-individual variability was detected.	A	ø
(80)	OLT	Autistic children (*N* = 30)	Probiotic supplementation *(Lacto.* spp., *Bifid. longum)*	ATEC scores, GM composition, clinical gastrointestinal symptoms using a structured assessment, anthropometric parameters	3 months	BW decreased significantly, ATEC scoresimproved, and gastrointestinal symptoms severity was reduced vs. baseline.	An increase in the *Bifid.* and *Lacto.* levels in the stool of these patients was observed. Small sample size.	C	ø
(81)	Case report	A 12-year-old boy, diagnosed with ASD and severe cognitive disability	Probiotic (a mixture of ten species- *Bifid.* spp., *Lact.* spp., *Strep.* spp.) as an add-on	ADOS-2	4 weeks, FU visit at week 8	The severity of gastrointestinal symptoms decreased, and the core symptoms of ASD also significantly improved after a few weeks of probiotic administration. The Social Affect” dimension scores of the ADOS improved after eight weeks, and the favorable evolution continued after another two months.	Repetitive behaviors did not improve during probiotic administration.	D	-
(82)	Case-control study	Children with ASD (*N* = 10), siblings (*N* = 9), and healthy children (*N* = 10)	Probiotic supplement *(Lacto, Bifid., Strep.)*	GM composition, CARS scores, gastrointestinal symptoms (parents’ reports)	16 weeks	The *Bacteroidetes/* *Firmicutes* ratio normalized, and the representation of *Desulfovibrio* spp. and *Bifid* spp. improved also, in medication-free children with ASD.	The *Bacteroidetes/Firmicutes* ratio was lower in ASD children vs. healthy controls.	C	-
**Attention-deficit/hyperactivity-disorder**									
(85)	DBRCT	Children and adults with ADHD (*N* = 182)	A synbiotic *(Pediococcus pentasaceus, Lact.* spp. + inulin, β-glucan, pectin, and resistant starch) vs. placebo as an add-on	ADHD symptoms severity determined through validated scales	9 weeks	The synbiotic improved sub-threshold ASD manifestations in children and emotion regulation in goal-oriented behaviors in adults.	A high baseline sVCAM-1 level in adults was associated with significant improvement in emotion regulation. In children, it was associated with a reduction of the total score of autism symptoms and restricted, repetitive, and stereotyped behaviors. Concomitant medication may interfere with the effects of synbiotics.	A	+
(86)	OLT	Children with ADHD (*N* = 30)	Probiotics (*Bifid.* *bifidum)*	ADHD symptoms, BW, BMI, GM composition	8 weeks, FU at 12 weeks	During the treatmenty period, inattention and hyperactive/impulsive symptoms improved, while the GM composition changed, with *Firmicutes/Bacteroidetes* ratio significantly decreasing.	The BW and BMI of the participants increased during the trial. No control group, either a placebo or an active comparator, and a small sample overall. ADHD symptoms were subjectively determined.	C	-
(87)	Longitudinal, observational trial	Healthy infants (*N* = 75)	Probiotics *Lacto.* *rhamnosus*) vs. placebo	GM composition, clinical evaluation	6 months, 13 years FU	ADHD or Asperger syndrome was diagnosed in significantly more children who received placebo.	The number of the *Bifidobacterium* in the GM during the first six months of life was significantly lower in children who subsequently developed psychiatric disorders. High rates of drop-out, multiple factors that might influence the results and have not been controlled for.	D	-

AD, Alzheimer’s disease; ADHD, attention-deficit/hyperactivity disorder; ADOS, Autism Diagnostic Observation Scale; ADT, antidepressants; ASD, autism spectrum disorders; ATEC, Autism Treatment Evaluation Checklist; Bifid., Bifidobacterium; BAI, Beck Anxiety Inventory; BD, bipolar depression; BDI, Beck Depression Inventory; BDNF, brain-derived neurotrophic factor; BW, body weight; CARS, Childhood Autism Rating Scale; Cl., Clostriodioides; DASS-21/42, Depression Anxiety Stress Scale-21/42; DBRCT, double-blind, randomized controlled trial; EPDS, Edinburgh Postnatal Depression Scale; EPL, Everyday Problem List; FMT, fecal microbiota transplantation; FU, follow-up; GAD, generalized anxiety disorder; GDS, Geriatric Depression Scale; GM, gut microbiota; HADS, Hospital Anxiety and Depression Scale; HDRS-17, Hamilton Depression Rating Scale-17; Ile, isoleucine; JMCIS, Japanese version of the MCI Screen; Lacto., Lactobacillus; LIDSR, Leiden Index of Depression Sensitivity-Revised; MAAS, Maternal Antenatal Attachment Scale; MADRS, Montgomery-Asberg Depression Rating Scale; MCI, mild cognitive impairment; MDD, major depressive disorder; MCP-1, monocyte chemotactic protein-1; MIP-1β , macrophage inflammatory protein-1 beta; MPAS, Maternal Postnatal Attachment Scale; OLT, open-label trial; OQR, Overall quality rating; PANSS, Positive and Negative Syndrome Scale; PES, Pregnancy Experience Scale; PHQ-9, Patient Health Questionnaire; PRAQ-R, Pregnancy-Related Anxiety Questionnaire-Revised; RANTES, regulated on activation, normal T cell expressed and secreted; RBAN, Repeatable Battery for the Assessment of Neuropsychological Status; RCT, randomized controlled trial; SAE, serious adverse event; SCHZ, schizophrenia; SSRIs, selective serotonin reuptake inhibitors; STAI-6, State-Trait Anxiety Inventory-6; sVCAM-1, Circulating Vascular Cell Adhesion Molecule-1; Strep., Streptococcus; Trp, tryptophan; YMRS, Young Mania Rating Scale.

The quality of the research is presented in Supplementary Table 2. Most of the results were gathered from the research of moderate (*n* = 18) or high (*n* = 20) quality, but low-quality reports were also identified (*n* = 5). The majority of the analyzed data originated in primary reports (i.e., clinical and preclinical studies, cohort studies, and case reports) (*n* = 34). Still, secondary reports were also identified and assessed (i.e., reviews or meta-analytic research) (*n* = 9).

### 4.1. Major depressive disorder and bipolar disorders

In most trials dedicated to patients diagnosed with depressive disorders, a decreased α-diversity of the GM has been found vs. the general population, and the family *Ruminococcaceae*, genus *Roseburia*, and *Faecalibacterium* were especially affected (19). However, it is yet impossible to certainly attribute this lower diversity of GM to a vulnerability toward or to an effect of depression (20). Another important aspect is the inconsistent reporting of this phenomenon across trials in all individuals with depression (19). GM is also involved in synthesizing monoaminergic neurotransmitters and BDNF, which are presumed to be involved in the pathogenesis of mood disorders (20).

According to a systematic review (*n* = 13 trials), probiotics containing *Bifidobacterium* and/or *Lactobacillus* spp. may exert a positive effect on depressive symptoms, although this conclusion is not unanimously supported (seven trials agreed on the beneficial result, while six did not find significant improvement in depressive scores during probiotic supplementation) (19).

In a meta-analysis, probiotic use in pregnancy was associated with favorable results, but these were not statistically significant (*n* = 2 randomized controlled trials, *N* = 545 participants) (31). The sub-population which benefited most from the addition of probiotics was represented by pregnant women with a lower score for depression (31). Still, a randomized, placebo-controlled trial explored the effects of *Lactobacillus rhamnosus* in pregnant women and during the postpartum period on symptoms of depression and anxiety (*N* = 423 women, recruited at 14–16 weeks of gestation) (21). The participants received this probiotic or a placebo up to 6 months postpartum (21). Participants receiving the active intervention had significantly lower depression and anxiety severity than those in the placebo group (21).

A meta-analytic research targeting the effects of psychobiotics on the severity of depressive symptoms in the adult population vs. an inactive comparator or placebo identified 50 studies that supported statistically significant benefits for pre, pro, or synbiotics (22). A favorable evolution was observed in individuals with and without depression (22). However, the authors considered the trials included in this analysis as having heterogeneous quality and likely publication bias (22). It is also worth mentioning that individual studies rarely reported major benefits, probably because the monitoring of depressive symptoms was considered only a secondary outcome (22).

An 8 weeks open-label trial evaluated the effects of probiotics (*Clostridioides butyricum* MIYAIRI 588, 60 mg/day) as add-ons to antidepressants (mainly selective serotonin reuptake inhibitors and duloxetine) in adults presenting major depressive disorder (MDD) (*N* = 40 participants) (23). The improvement of depressive symptoms was significant on all scales- Hamilton Depression Rating Scale (HDRS-17), Beck Depression Inventory (BDI), and Beck Anxiety Inventory (BAI) (23). At the final study visit, 70% of the participants were responders, while 35% were remitters (23). The overall tolerability was good, and no serious adverse events were reported (23).

A large cross-sectional U.S. population-based study evaluated the odds of developing depression in adult subjects who consumed probiotics versus the general population (*N* = 18,019 participants), and a Patient Health Questionnaire (PHQ-9) score of more than 10 was used to establish the existence of depression (24). The probiotic foods included in this analysis were yogurt, kefir milk, buttermilk, and kimchi, and 152 different probiotic supplements were also included (24). The analysis suggests that individuals who consumed probiotics had a lower risk for depression, but after data adjustment, the effect was no longer significant (24).

In a randomized trial, 110 patients with depression received a probiotic (*Lactobacillus helveticus* and *Bifidobacterium longum*), a prebiotic (galactooligosaccharide), or an inactive product during 8 weeks (25). Depressive symptoms improved in patients undergoing probiotic supplementation, and at the end of the study, the BDI scores decreased significantly vs. placebo and prebiotic (25).

A trial that enrolled 79 participants with mood symptoms self-evaluated as being of at least moderate severity, randomly assigned to a probiotic (*Lactobacillus helveticus* and *Bifidobacterium longum*) or an inactive compound, in a double-blind, 8 weeks trial (26). The results were not supportive of the efficacy of probiotics vs. placebo on any outcome psychological measures or biomarkers (26). At the endpoint, 23% of the subjects randomized on probiotics were responders, according to the Montgomery-Asberg Depression Rating Scale (MADRS) scores evolution vs. 26% in the placebo group (26).

A double-blind, placebo-controlled trial enrolled 40 MDD patients, randomly assigned to probiotic supplementation (*Lactobacillus acidophilus, Bifidobacterium bifidum*, and *Lactobacillus casei*) or placebo for 8 weeks (27). The improvement of BDI scores was significantly superior to the placebo after 8 weeks, and insulin, HOMA-IR, and serum hs-CRP levels also diminished in participants receiving active therapy vs. placebo (27). The glutathione levels increased significantly in patients receiving probiotics (27).

In a randomized trial, 38 patients with type 1 bipolar disorder (BD) received probiotics (*Bifidobacterium bifidum, lactis, langum*, and *Lactobacillus acidophilus*, 1.8 × 10^9^ CFU/capsule) or placebo, and they were monitored for 2 months (28). At the last study visit, no significant changes were observed on the Young Mania Rating Scale (YMRS) or HDRS between groups, although a trend toward superiority in participants treated with probiotics was reported (28).

A triple-blind, placebo-controlled trial enrolled 71 individuals who were randomized on either a probiotic or placebo for 8 weeks (29). The active intervention correlated with a significantly higher reduction in cognitive functioning vs. placebo, but probiotics did not induce any significant modification of the gut microbiota in depressed patients (29). All participants presented at endpoint improvements in depressive symptoms, which raises the possibility of non-specific therapeutic factors involved in this phenomenon (i.e., frequent visits to the clinic) (29).

Synbiotic (15 g prebiotics, 5 g probiotic- *Lactobacillus acidophilus, Bifidobacterium bifidum, B. lactis, B. longum*, 2.7 × 10^7^ CFU/g each) or probiotic (5 g probiotics as mentioned previously + 15 g placebo) supplementation in 75 hemodialysis-undergoing patients was compared with placebo (20 g maltodextrin) for 3 months (30). Synbiotics or probiotics were superior to placebo regarding the improvement of the Hospital Anxiety and Depression Scale (HADS)–Depression subscale score in patients with initial depressive symptoms, compared to placebo and probiotics interventions (30). All participants improved their depressive severity scores compared to placebo during synbiotics administration (30).

In conclusion, based on mostly moderate and high-quality data derived from eight clinical trials, one population study, and three systematic reviews/meta-analyses, the use of probiotics was associated with more positive than negative results, while prebiotics administration was not supported. The majority of the trials evaluated were short-term, included a low number of patients, the intervention was heterogenous, and the population was also very diverse (e.g., the severity of depressive manifestations at baseline, the type of mood disorder, the age).

### 4.2. Anxiety disorders

Anxiety disorders are a heterogeneous group and different reports about GM changes in patients diagnosed with this pathology exist in the literature. In one such study, generalized anxiety disorder (GAD) patients presented a significant difference in microbiota diversity and richness vs. healthy controls, with *Fusicatenibacter* and *Christensenellaceae* spp. being significantly lower vs. controls (65). Systematic reviews found inconsistencies in the reported α and β diversity in patients with anxiety disorders, but an increased abundance of proinflammatory species and lower short-chain fatty acid (SCFAs)-synthesizing species were more frequently signaled across studies (66).

The results of a meta-analysis (*n* = 2 trials, *N* = 543 patients) confirmed that the administration of probiotics (*Lactobacillus* spp*., Bifidobacterium* spp.) during pregnancy decreased the severity of anxiety (assessed on the STAI-6 questionnaire) when compared to placebo, although this improvement was moderate if more rigorous criteria were used (31).

Pregnant women (*N* = 40) with low-risk pregnancies and severe depressive and/or anxiety symptoms received a probiotic (*Bifidobacterium bifidum, lactis* spp*., Lactobacillus acidophilus, brevis, casei, salivarius, Lactococcus lactis* spp.) or placebo, starting from 26–30 weeks of gestation until delivery, in a randomized, double-blind controlled trial (32). After 8 weeks of treatment, no major change was found in the efficacy outcomes (Edinburgh Postnatal Depression Scale, Leiden Index of Depression Sensitivity-Revised, Pregnancy-Related Anxiety Questionnaire-Revised, State-Trait Anxiety Inventory, Pregnancy Experience Scale, Everyday Problem List, The Maternal Antenatal Attachment Scale, and The Maternal Postnatal Attachment Scale) when the two groups were compared (32). The number of adverse and serious adverse events was similar in the two groups (32).

In a previously mentioned, triple-blind, randomized, placebo-controlled trial, the probiotic intervention did not induce any significant modification of the GM in patients presenting anxiety symptoms associated with depression (*N* = 71 participants) (28).

A systematic review (*n* = 12 studies) found that probiotics (*Bifid., Lact., Strep. salivarius/termophilus, Cl. butyricum*, and *Lactococcus* spp.) may be useful in the management of elevated stress, anxiety, or depression in adults (33). Probiotics have been found in the reviewed controlled and uncontrolled trials to reduce depression (*n* = 6 studies) and anxiety (*n* = 2 studies) (28). It should be noted that the same review found five trials that did not report any improvement in anxiety or depression vs. placebo.

In a randomized trial, 150 healthy volunteers received probiotic oral suspension (3 g/day, containing *Streptococcus thermophiles, Bifidobacterium animalis subsp. lactis, Bifidobacterium bifidum, Lactobacillus bulgaricus, L. lactis, L. acidophilus, L. plantarum, L. reuteri*) or placebo for 3 months, and the HAMA total score was significantly reduced in the active vs. control group (34). The carriers of IL-1β rs16944 single nucleotide polymorphism (related to high proinflammatory cytokine levels, depression, and neurodegenerative diseases) presented a moderate risk of having anxiety at baseline (43 vs. 11.4% in non-carriers), but the administration of probiotics helped in decreasing the HAMA score in this subgroup, while in the non-carriers the effect of probiotics was not significant (34).

In a randomized trial, 48 patients without current psychotropic treatment, diagnosed with generalized anxiety disorder, received probiotics (18 × 10^9^ CFU *Bifidobacterium longum, B. bifidum, B. lactis*, and *Lactobacillus acidophilus*) or placebo, administered in combination with 25 mg sertraline for 8 weeks (35). The efficacy of sertraline + probiotic intervention was superior to placebo on the anxiety symptoms, according to the evolution of the scores on the HAMA and State-Trait Anxiety questionnaires, but the reported quality of life was similar in the two groups at the endpoint (35).

In a previously mentioned trial, the administration of synbiotic or probiotic supplementation in patients undergoing hemodialysis was compared with a placebo, and no superiority of the active intervention was detected at the endpoint regarding the improvement of the HADS–Anxiety subscale score (30). However, synbiotics significantly improved these scores vs. baseline values in all subjects, and also in those participants with depression at baseline (30).

The administration of probiotics was associated with improvements in panic, neurophysiological anxiety, negative affect, and worry in a group of healthy students participating in a double-blind, placebo-controlled trial (36). Patients with a high level of distress had more dimensions improved (BAI, Positive and Negative Affect Schedule, Penn State Worry Questionnaire, Negative Mood Regulation, Anxiety Control Questionnaire- revised) vs. those with normal distress, signaling a ceiling effect (36).

A randomized, double-blind, placebo-controlled study included 111 adults with moderate stress levels who received probiotics (*Lactobacillus plantarum* DR7) or a placebo for 3 months (37). The probiotics significantly decreased symptoms of stress and anxiety, starting from week 8 vs. placebo, as observed during the monitoring of the DASS-42 questionnaire scores (37). Plasma cortisol and pro-cytokines levels were reduced in subjects receiving probiotics, while cognitive and mnestic functioning improved in healthy, mature subjects vs. placebo and young adults (37).

In conclusion, based on nine reports identified in the literature, consisting of seven trials and two reviews of moderate and high quality, the effect of probiotics in decreasing anxiety manifestations was supported by several good-quality studies but invalidated by others. Synbiotics were not associated with significant results in this population. The overall tolerability of probiotics was good, but very few studies reported on this dimension.

### 4.3. Schizophrenia spectrum disorders

Therapeutic approaches to SSD are limited to antipsychotics with different metabolic or neurological adverse events, while psychotherapy and other types of explored interventions have very limited benefits (3, 67, 68). Therefore, new treatments for these patients are necessary in order to improve their prognosis and overall functionality. Alterations in metabolites (e.g., SCFAs), changes in neurotransmission (e.g., GABA, glutamate, serotonin) and neurotrophic factors, and immunity impairments (e.g., altered blood T-cell numbers) have been suggested as intermediary stages between GM dysbiosis and the onset of SSD (3). Still, the correlations between specific GM changes and schizophrenia have not yet been validated, and antipsychotics have been associated with the potential to cause metabolic dysfunctions *via* microbiome alteration (69). A study using germ-free C57BL/6J mice showed that olanzapine potentiated a change toward a diathesis vulnerable to obesity in GM (64). Also, olanzapine has antimicrobial activity *in vitro* against certain species of bacteria within the GM (e.g., *Enterococcus faecalis, Escherichia coli*) (64). However, in a GM analysis of 90 medication-free patients with schizophrenia vs. 81 controls, it was observed that the first group presented differences in SCFAs, and neurotransmitters degradation or synthesis; therefore, at least some changes exist prior to the onset of the antipsychotic treatment in schizophrenia (70). GM in schizophrenia may be associated with neurostructural changes, psychopathology severity, subclinical inflammatory processes, and higher cardiovascular risk (71). Germ-free mice receiving fecal microbiome transplants (FMT) from patients with SSD had lower glutamate and higher glutamine/GABA concentrations in the hippocampus vs. healthy controls (72). The authors of the same study concluded that schizophrenia-like behaviors might be related to hypo-glutamatergic function (72).

Biotherapeutic products, i.e., probiotics, prebiotics, and polyphenols, have been hypothesized as potential add-ons to the antipsychotic treatment in patients with SSD (73). The positive influence of these products on BDNF serum levels might represent the factor behind the improvement of clinical evolution in this population (73).

A meta-analysis of trials with psychobiotics, antibiotics, and antimicrobials as add-ons in schizophrenia (*n* = 28 studies) did not report significant improvements using probiotics (only one trial met the inclusion criteria) vs. placebo on the negative symptoms of schizophrenia (38). One study included in the same meta-analysis detected a trend toward efficacy vs. placebo when probiotics were combined with vitamin D (38). The overall tolerability of the explored add-on agents was similar to that of the placebo (38).

The supplementation of the current treatment in patients diagnosed with chronic schizophrenia with probiotics (*Lactobacillus rhamnosus* GG, *Bifidobacterium animalis* Bb12) vs. placebo for 14 weeks (*N* = 31 vs. 27 participants) led to the significant reduction of von Willebrand factor and increased borderline significant the MCP-1 (monocyte chemotactic protein-1), BDNF, RANTES, and MIP-1β (macrophage inflammatory protein-1β) levels (39). A distinct analysis showed that probiotics might exert their effects by regulating immune cell function and intestinal epithelial cell activity (39).

Outpatients diagnosed with schizophrenia (*N* = 65) who presented moderately severe psychotic manifestations were distributed randomly to 14 weeks of double-blind add-on probiotic (*Lactobacillus rhamnosus* GG and *Bifidobacterium animalis* Bb12) or placebo (40). The comparative analysis of the Positive and Negative Syndrome Scale (PANSS) total scores evolution did not detect differences between groups, but patients treated with probiotics developed less frequently severe bowel difficulty during the trial (40).

Another open-label trial enrolled 29 outpatients with schizophrenia who received probiotics (*Bifidobacterium breve* A-1) for 1 month, with a follow-up visit after another 4 weeks (41). Both the HADS total score and the mood scores on PANSS improved after 4 weeks, and 12 patients were considered responders (HADS reduction of more than 25%) at the endpoint (41). Responders also presented fewer negative symptoms and a more significant presence of *Parabacteroides* in the GM vs. non-responders (41). TRANCE and the expression of the IL-22 were significantly higher at 4 weeks after baseline in patients with a favorable response to the intervention (41).

Vitamin D3 (50,000 UI every 2 weeks) and probiotics (8 × 10^9^ CFU/day) supplementation vs. placebo in 60 patients with chronic schizophrenia, administered for 12 weeks, were compared in a randomized, placebo-controlled trial (42). The total and general PANSS scores improved significantly after 12 weeks, and the total antioxidant capacity also increased vs. placebo (42). Malonaldehyde levels and hs-CRP levels decreased, and fasting plasma glucose, insulin concentration, triglycerides, and total cholesterol levels were reduced vs. placebo (42).

In conclusion, based on the results of five reports, i.e., four clinical trials and one systematic review, mostly of moderate quality, the recommendation for the administration of the probiotics in SSD could not be supported. However, several data about the potential benefits of this intervention on SSD-associated mood symptoms are encouraging, and the association of probiotics with vitamin D deserves more exploration.

### 4.4. Substance use disorders

The excessive use of alcohol may affect GM in human and animal models, leading to a dysbiosis that can represent an essential link in the pathogenesis of alcohol use disorders (AUD). Most of the data regarding this subject are derived from animal studies, which showed a connection between chronic alcohol use and increased oxidative stress, higher intestinal permeability to different bacteria-produced toxic factors, and the onset of alcoholic hepatitis (2, 74). Increased dysbiosis may determine systemic inflammation and endotoxemia, as well as specific organ pathologies, supporting, at a theoretical level, the usefulness of a probiotic or synbiotic modulation of the GM as prophylactic measures or therapeutic interventions in AUD (2).

In a preclinical model of chronic-binge alcohol exposure (CBAE), the addition of a synbiotic product (consisting of a butyrate-producing and anti-inflammatory commensal bacteria + a butyrate-yielding prebiotic) was explored from the perspective of GM composition changes and hepatocyte lesions (43). In C57BL/6 female mice who received CBAE for 10 days, the GM decreased its abundance and diversity, and the hepatocytes were more damaged than in mice receiving gavage with saline solution vs. synbiotic (43). The synbiotic administered in mice exposed to alcohol use reduced the negative effects on the GM and liver endothelial barrier integrity (43).

Another study conducted by the same team showed the superiority of the synbiotic administration (*Faecalibacterium prausnitzii* + potato starch) by oral gavage vs. fecal slurry (fecal pellets) in C57BL/6 mice undergoing 10 days of chronic binge-eating alcohol when hepatic inflammation (TNF-alpha) and oxidative stress (4-HNE) were measured (44). Also, this study showed a decreased hepatic steatosis induced by alcohol exposure if synbiotics were concomitantly administered (44).

Another team of researchers demonstrated on male Wistar rats receiving either a normal liquid diet ± synbiotic or an ethanol liquid diet ± synbiotic supplementation for 3 months, that the addition of a synbiotic may reduce the plasma endotoxin, hepatic triglyceride, and TNF-α levels, and raise the hepatic IL-10 concentration (45).

In conclusion, the results of the three preclinical studies of moderate quality there is a possibility that probiotics may be of interest to human research of AUD in the near future.

### 4.5. Neurocognitive disorders

Changes in the GM can be considered between the potential pathogenic causes for the onset of neurocognitive disorders, for example, Alzheimer’s dementia (75). In a study, fecal samples from patients with Alzheimer’s disease and age-matched healthy controls were compared, and differences in GM were detected (e.g., *Bacteroides, Actinobacteria, Ruminococcus, Lachnospiraceae, Selenomonadales*) (76). Another study with a similar methodology reported a higher concentration of *Bifidobacterium, Sphingomonas, Lactobacillus*, and *Blautia* in patients with neurocognitive disorders, while *Odoribacter, Anaerobacterium*, and *Papillibacter* were reduced (77).

According to the results of a systematic review targeting trials dedicated to the effects of psychobiotics or FMT on cognitive functioning (*n* = 23 articles), probiotic supplementation improved the primary outcome (78). Most of the trials that enrolled healthy subjects communicated significant positive effects of probiotics in more than one performed cognitive task (78). In patients with cognitive impairments of different causes (Alzheimer’s disease, hepatic encephalopathy, HIV-infected individuals, MDD, Parkinson’s disease) the same adjuvants were associated with multiple favorable results on different cognitive tasks (78).

A meta-analysis dedicated to the effectiveness of probiotics and synbiotics on cognitive functioning in patients with dementia included three randomized controlled trials (*N* = 161 patients with Alzheimer’s disease) (46). *Lactobacillus* and *Bifidobacterium* strains were not associated with beneficial effects on cognitive functioning when used as probiotic supplements (46). The quality of data was rated as very low for this outcome, but the probiotics improved plasma levels of triglycerides, VLDL, insulin resistance, and plasma malondialdehyde (46).

Probiotic-fermented milk supplementation (2 ml/kg/day, kefir synbiotic) for 3 months was investigated in individuals with Alzheimer’s disease, in an open-label, uncontrolled trial (*N* = 16 participants) (47). Improvements in mnestic, visual-spatial, abstraction, executive and language functioning were observed, and the level of several inflammatory cytokines and oxidative stress markers decreased (47). Outcomes related to oxidative stress were also improved at the end-point (47).

In a study that enrolled 49 elders, synbiotic supplementation was compared to placebo, and the results support a favorable change in both groups regarding the percentage of body fat, TNF-alpha level, and serum diamine-oxidase (48). The IL-6, Geriatric Depression Scale-15 items version (GDS-15) score, and Mini-Mental State Examination (MMSE) score increased in both groups (48). IL-10 increased only during the synbiotic treatment, and lipopolysaccharide (LPS) decreased only in the placebo group (48). In conclusion, the effects of synbiotic vs. placebo on depressive symptoms and cognitive functioning were modest at 6 months in a group of apparently healthy elders (48).

In a randomized, controlled trial (*N* = 79 patients diagnosed with Alzheimer’s disease), selenium (200 μg/day) + probiotic (*Lactobacillus acidophilus, Bifidobacterium bifidum, Bifido bacterium longum*, 2 × 10^9^ CFU/day each) was compared to selenium as monotherapy (200 μg/day) or placebo for 3 months (49). Probiotic + selenium intake led to the reduction of the hs-CRP levels and an increase in the overall antioxidant capacity and total glutathione (GSH) vs. selenium as monotherapy or placebo (49). Also, lower insulin levels and HOMA-IR and higher QUICKI (quantitative insulin sensitivity check index) were associated with combined treatment vs. placebo or selenium monotherapy (49). Serum levels of triglycerides, VLDL, LDL, and total-/HDL-cholesterol, were significantly reduced by this combination of selenium and probiotics vs. selenium as monotherapy and placebo (49).

In a randomized, placebo-controlled trial, elders diagnosed with MCI (*N* = 80 otherwise healthy participants) received either a daily probiotic (*Bifidobacterium breve* A1, 2 × 10^10^ CFU) or a placebo for 4 months (50). The cognitive functioning improved significantly in individuals using probiotics vs. placebo after 16 weeks of treatment, using a structured assessment scale (Repeatable Battery for the Assessment of Neuropsychological Status, RBANS) (50). Immediate memory, visuospatial/constructional, and delayed memory were significantly improved, as well as the global cognitive score, at the end-point (50).

Another randomized, placebo-controlled trial explored the effects of probiotics (*Bifidobacterium bifidum* and *Bifidobacterium longum*) on cognition and mood in older adults (*N* = 63 healthy participants) for 12 weeks (51). At the end-point, the relative abundance of GM species involved in inflammation pathogenesis decreased significantly in patients undergoing probiotic treatment, while the same patients presented greater improvement in mental flexibility tests and stress scores vs. placebo (51). Probiotics increased serum BDNF levels and changed the composition of the GM (mainly *Eubacterium* and *Clostridioides* representation) (51).

In a randomized, placebo-controlled trial, *Bifidobacterium breve* A1 supplementation was added for 3 months in 121 elderly individuals with cognitive complaints (52). The neuropsychological tests (RBANS, MMSE) scores supported a significant improvement in both groups, without differences between interventions (52). Immediate memory was, however, more improved under probiotics vs. placebo, both according to the RBANS and MMSE tests, but only in subjects with low RBANS scores at baseline (52). The tolerability of probiotic supplementation was good during the entire period of the study (52).

In conclusion, the results of six clinical trials and one meta-analysis, mostly of moderate quality, support the necessity of further exploration for probiotics (eventually associated with selenium) and synbiotics in patients with MCI and neurocognitive disorders. Although currently there is not enough data to recommend their use in this population, there are several encouraging results, both on specific cognitive dimensions, and on modification of the GM composition, that could reduce inflammation and oxidative stress. The tolerability of psychobiotics, assessed in very few reports, was good.

### 4.6. Eating disorders

Eating disorders have been associated with high risks for overall health status, quality of life, and general functionality (4, 79). Dietary, probiotics/prebiotics/synbiotics administration, and FMT have been conceptualized as possible interventions for patients diagnosed with anorexia nervosa (AN) (80). The modulation of weight gain in these patients’ recovery involves GM changes, but the specific interaction between these two variables has not been elucidated (80). An analysis of the GM composition and diversity in AN patients vs. healthy controls revealed higher interindividual variation in the first group, suggesting altered GM functioning (81). Lower levels of serotonin, GABA, dopamine, butyrate, and acetate in AN patients’ feces were detected when compared to healthy controls (81). A longitudinal analysis of AN patients’ symptoms, BMI, and GM composition and metabolites, did not support a correlation between the BMI increase/symptoms improvement, on the one hand, and short-chain fatty acids, neurotransmitters profile, and GM composition, on the other (81).

Modulation of GM was investigated as an adjuvant in the treatment of obesity. Colonic dysbiosis may create a favorable terrain for neuroinflammation and behavioral changes, while obesity may be correlated with an important accumulation of persistent organic pollutants (50). Therefore, targeting GM could enhance the body detoxification process, and pre/pro/synbiotics could be helpful in this direction (82). A review of the randomized trials targeting the efficacy of psychobiotics in obese patients (*n* = 28 trials) suggests the prebiotics have a neutral impact on body weight, decreased fasting and postprandial glucose, improved insulin sensitivity, and lipid profile, with the possible reduction of inflammatory markers (53). The same source showed that probiotics have significant minor effects on body weight and metabolic parameters, and the changes in GM were not constantly reported during pre or probiotic use (53).

In patients with obesity (*N* = 101 participants), the analysis of GM showed a decrease in *Akkermansia* and *Intestimonas* distribution and an increase in *Bifidobacterium* and *Anaerostipes* (63). The same study showed low affect balance, impairments in inhibition and self-regulation, and increased emotional and external eating in patients with binge eating disorder (BED) vs. controls (63).

In conclusion, the data is yet inconclusive for the support of psychobiotics use in specific eating disorders.

### 4.7. Autism spectrum disorders

Functional gastrointestinal disorders are frequently diagnosed as a comorbidity in cases of autism spectrum disorders (ASD), and a common origin in gut dysbiosis has been suggested for these disorders (83). Children with ASD are estimated to present a four times higher risk of experiencing gastrointestinal symptoms vs. children without ASD (55). Therefore, pre- and probiotic supplementation represents possible useful interventions in children with ASD, but the findings to support this hypothesis are rather limited, with potential benefits in reducing gastrointestinal discomfort, improving ASD behaviors, changing GM composition, and reducing the inflammatory diathesis (83). Administration of probiotics containing *Lactobacillus* and *Bifidobacteria* strains may favor gastrointestinal and behavioral symptoms in ASD patients with gastrointestinal disturbances (84).

A systematic review (*n* = 14 articles) investigated the efficacy of different interventions focused on GM modulation in ASD patients, with negative results for probiotic studies, while prebiotics and synbiotics may be efficacious in improving specific behavioral symptoms (54). Another narrative review (*n* = 5 articles, *N* = 117 participants) concluded that the available data are of poor methodological quality and allow for multiple confounding factors (e.g., diet, concomitant medication, different dosages or strains administered) (55). However, probiotics may be helpful for ASD patients, and they may alter the fecal microbiota or urine metabolites in a beneficial direction while reducing the ASD symptoms severity (55).

The administration of a prebiotic (*Lactobacillus plantarum* WCSF1) in 22 ASD children during a double-blind crossover trial with a 12 weeks duration led to significant differences in behavioral scores (assessed on Developmental Behavior Checklist) at the end-point vs. baseline (56). Probiotics also led to a substantial increase of *Lactobacilli* and *Enterococci* groups while significantly decreasing *Clostridioides* cluster XIVa vs. placebo (56). A very high dropout rate was reported, indicating the need to interpret these results with caution. Also, a high inter-individual variability involves the necessity to enroll more homogenous groups of patients with ASD (56).

After probiotic supplementation (each gram containing 100 × 10^6^ CFUs of *Lactobacillus acidophilus*, *Lactobacillus rhamnosus*, and *Bifidobacteria longum*) in a study that enrolled 30 autistic children (5–9 years old), there was reported a higher level of *Bifidobacteria* and *Lactobacilli* in the stool (57). Also, their body weight decreased significantly, the Autism Treatment Evaluation Checklist (ATEC) scores improved, and gastrointestinal symptoms severity decreased vs. baseline (57).

In a single case report, a 12 years-old boy diagnosed with ASD and severe cognitive disability received 4 weeks of an add-on mixture containing 10 probiotics (VSL#3) (58). The diet was preserved during the 8 weeks of monitoring (58). The severity of digestive manifestations decreased, and the core symptoms of ASD also significantly improved after a few weeks of probiotic administration (58). The “Social Affect” dimension scores of the Autism Diagnostic Observation Scale (ADOS) improved after 8 weeks, and the favorable evolution continued after another 2 months (58).

The administration of a probiotic diet supplementation (“Children Dophilus,” containing *Lactobacillus, Bifidobacteria*, and *Streptococcus*) three times daily for 12 weeks normalized the *Bacteroidetes/Firmicutes* ratio, the representation of *Desulfovibrio* spp. (a suspected pathogenetic factor of autism) and *Bifidobacterium* spp. in feces of medication-free ASD children (*N* = 10) (59). These patients had at baseline a significantly lower *Bacteroidetes/Firmicutes* ratio, an increased representation of the *Lactobacillus* genus, and a tendency to increase *Clostridioides* class 1 abundance vs. the control group (59).

In conclusion, based on data derived from six reports of mostly moderate and low quality, consisting of two clinical trials, one case-control study, one case report, and two reviews, probiotics may be beneficial for associated gastrointestinal manifestations in patients with ASD. Regarding the effects of psychobiotics on core ASD manifestations, the data reviewed were inconclusive.

### 4.8. Attention-deficit/Hyperactivity disorder

Attention deficit hyperactivity disorder in children has been associated with a higher representation of *Bacteroidaceae* and *Neisseriaceae*, which may cause a significant decrease in GM heterogeneity (85). Neuroinflammation in ADHD patients, abnormal activation of microglia, and altered proportion between pro- and anti-inflammatory cytokines may alter the maturation of the prefrontal cortex and the neurotransmission systems, increasing the risk for ADHD onset (86).

A synbiotic was added to children and adults with ADHD (*N* = 182) for 9 weeks in a randomized, placebo-controlled trial, and the results were not significantly different in the primary outcome (ADHD symptoms severity) (60). Synbiotic 2,000 decreased sub-threshold ASD manifestations (restricted, repetitive, and stereotyped behaviors) in children and had a favorable impact on emotion regulation in goal-oriented behavior in adults (60). If a high level of sVCAM-1 were detected at baseline, in adults, the synbiotic significantly improved emotion regulation (60). In children, this product reduced the overall severity of autism symptoms and the sub-domains of ASD behaviors (60).

Probiotics supplements with *Bifidobacterium bifidum* (Bf-688) 5 × 10^9^ CFUs/day were administered for 8 weeks in an open-label trial that enrolled 30 children diagnosed with ADHD (61). During the treatment period, inattention and hyperactive/impulsive symptoms improved, while the GM composition changed, with *Firmicutes/Bacteroidetes* ratio significantly decreasing (61). Also, the weight gain and BMI of the participants increased during the trial (61).

An interesting study followed longitudinally for 13 years a group of 75 infants randomized to receive *Lactobacillus rhamnosus* GG or a placebo during their first 6 months of life (62). At the end of the monitoring period, ADHD or Asperger syndrome was detected in 17% of the subjects in the placebo group vs. none in the probiotic-receiving group (62). The number of the *Bifidobacterium* in the GM during the first 6 months of life was significantly lower in children who subsequently developed psychiatric disorders (62).

In conclusion, based on two clinical trials and one cohort study, of heterogenous quality, synbiotics may improve associated autistic symptoms, and probiotics may decrease inattention and hyperactive/impulsive symptoms. Also, a potential prophylactic effect of probiotics in children, if administered early in life, was detected, but this conclusion is based on very limited support.

## 5. Conclusion

Regarding the main objective of this review, the data supporting the efficacy of psychobiotic, primarily probiotics, as adjuvants in the treatment of psychiatric disorders is mixed. According to mostly moderate and high-quality data derived from primary (*n* = 9) or secondary (*n* = 3) reports, the use of probiotics was associated with more positive than negative results, while prebiotics administration was not supported in the treatment of *uni- or bipolar depression*. There are some limitations of these trials because most of them were conducted on short-term, included a low number of patients, the intervention was heterogenous, and the population was also very diverse (e.g., the severity of mood manifestations at baseline, the type of depression, or the age). Based on primary (*n* = 7) and secondary reports (*n* = 2), of moderate and high quality, the effect of probiotics in decreasing *anxiety manifestations* was controversial, and the use of synbiotics did not lead to significant results in this population. No conclusive results for the efficacy of probiotics in patients with *SSD* as adjuvant treatment could be found, according to primary (*n* = 4) or secondary (*n* = 1) reports, mostly of moderate quality. Based on the results of three primary reports of moderate quality, there is currently no support for the benefit of psychobiotics in patients with *SUD*. Primary (*n* = 6) and secondary (*n* = 1) reports, mostly of moderate quality, probiotics ± selenium and synbiotics deserve more exploration in patients with *MCI* and *neurocognitive disorders*. There is insufficient data yet to elaborate on the usefulness of psychobiotics in *specific eating disorders*, with only one secondary, high-quality report being reviewed. Based on data derived from four primary and two secondary reports of mostly moderate and low quality, probiotics may be beneficial for associated gastrointestinal symptoms in individuals with *ASD*. Synbiotics may be efficient in patients with *ADHD* for improving associated autistic symptoms, and probiotics may decrease inattention and hyperactive/impulsive symptoms, according to the results of three heterogeneous quality primary reports. The overall tolerability of probiotics was good, but only a minority of studies reported on this dimension.

The secondary objective, which referred to the possibility of formulating a clinical recommendation for the use of psychobiotics in specific psychiatric disorders, the reviewed reports did not currently support such a strategy. The most promising data are for the patients with mood disorders, who may benefit from the administration of probiotics, but there is still much heterogeneity in the products used to enable a specific therapeutic add-on recommendation. Probiotics may be useful in patients with ASD (for associated symptoms, especially gastrointestinal) and ADHD (also for associated symptoms, but for core symptoms, too), but, again, it is too early to formulate specific recommendations.

Regarding new perspectives on the interplay between GM and psychiatric disorders, data in the literature reflect intense efforts to find different ways to modulate the microbiome in order to enhance stress resilience. Increasing resilience to stressors by influencing GM through diet has been explored in animal models of depression, cognitive impairment, Parkinson’s disease, ASD, and epilepsy (87). Anti-inflammatory effects mediated by the microbial metabolites of dietary fibers and polyphenols are considered responsible for the benefits of diet on GM (87). An increased abundance of diverse GM species able to produce SCFA, e.g., *F. prausmitzii, E. rectale, Roseburia*, and *A. mucinophilia*, has been associated with the use of the Mediterranean diet (87). Vagotomy has been reported to block depression-like phenotypes in rodents after FMT of the microbiome from depressed subjects, which involves a complex interplay between the GM, vagus nerve, stress resilience, and depression (88). Interventions aiming at the manipulation of GM during the first phases of development in order to prevent or decrease the effects of early-life stressors are still under investigation (89). This type of research could indicate the existence of epigenetic modulations through GM changes, which might open an entirely new perspective on stress resilience; this, in turn, could raise the possibility of increasing the chances of therapeutic and even prophylactic interventions for psychiatric disorders.

Although other literature reviews dedicated to this topic exist and were cited in the previous sections of this paper (18, 20, 22, 31, 33, 38, 54), the current research explored all the major psychiatric disorders both in adults, adolescents, and children, including primary and secondary reports, without restriction to the type of the psychobiotics administered. A meta-analysis targeting the effectiveness of probiotic supplementation in psychiatric disorders (*n* = 23 studies) concluded that probiotics might be useful in reducing depressive symptoms in a statistically significant proportion vs. placebo, but not in the case of schizophrenia, stress, and anxiety (90). These conclusions are similar to the current systematic review, stressing the potential beneficial role of probiotics in mood disorders. Even more, the previously cited meta-analysis concluded that parameters like the probiotic composition, the quality of ingested psychobiotics, and the trial length significantly modulate the results of the active intervention vs. placebo (90). Regarding the studies on prebiotics and synbiotics, the results of their administration in patients with psychiatric disorders were inconclusive, according to another systematic review (91). The need for more well-designed trials focused on specific probiotic strains, inter-individual GM variations, and more homogenous phenotypes of psychiatric disorders has been emphasized by other authors exploring this topic (91, 92).

Limitations of the review refer to the selection and assessment of the quality of data which was conducted by only one researcher, and to the limited duration of the primary reports which may prevent the observation of long-term effects of psychobiotics use. Also, the high heterogeneity of several psychiatric nosographic categories, e.g., mood disorders, anxiety disorders, or SSD, makes it difficult a signal detection of psychobiotics. Different interactions between pre-, pro-, or synbiotics and currently administered psychotropics is a difficult-to-eliminate bias factor.

Although the reviewed data could not be translated into clinical recommendations, there is enough evidence to grant further research, especially for the assessment of the efficacy of psychobiotics in patients diagnosed with mood disorders, ASD, and ADHD.

## Data availability statement

The original contributions presented in this study are included in this article/Supplementary material, further inquiries can be directed to the corresponding author.

## Author contributions

The author confirms being the sole contributor of this work, and has approved it for publication.

## Footnotes

1
www.clinicaltrials.gov
