# Supplementary material for: The current state of research for psychobiotics use in the management of psychiatric disorders–A systematic literature review
Source: Front Psychiatry. 2023 Feb 23;14:1074736. doi: 10.3389/fpsyt.2023.1074736 (PMC9996157; doi:10.3389/fpsyt.2023.1074736)
Supplement: Supplementary file 1 [file Data_Sheet_1.docx]

**Table 1. The PRISMA 2020 Checklist** [16]

| **Section and Topic** | **Item #** | **Checklist item** | **The location where an item is reported** |
| --- | --- | --- | --- |
| **TITLE** | | |  |
| Title | 1 | Identify the report as a systematic review. | Line 19 |
| **ABSTRACT** | | |  |
| Abstract | 2 | See the PRISMA 2020 for the Abstracts checklist. | Lines 15-38 |
| **INTRODUCTION** | | |  |
| Rationale | 3 | Describe the rationale for the review in the context of existing knowledge. | Lines 40-62 |
| Objectives | 4 | Provide an explicit statement of the objective(s) or question(s) the review addresses. | Lines 65-70 |
| **METHODS** | | |  |
| Eligibility criteria | 5 | Specify the inclusion and exclusion criteria for the review and how studies were grouped for the syntheses. | Lines 91-106 |
| Information sources | 6 | Specify all databases, registers, websites, organizations, reference lists, and other sources searched or consulted to identify studies. Specify the date when each source was last searched or consulted. | Lines 79-82 |
| Search strategy | 7 | Present the full search strategies for all databases, registers, and websites, including any filters and limits used. | Lines 78-88 |
| Selection process | 8 | Specify the methods used to decide whether a study met the inclusion criteria of the review, including how many reviewers screened each record and each report retrieved, whether they worked independently, and if applicable, details of automation tools used in the process. | N/A |
| Data collection process | 9 | Specify the methods used to collect data from reports, including how many reviewers collected data from each report, whether they worked independently, any processes for obtaining or confirming data from study investigators, and if applicable, details of automation tools used in the process. | N/A |
| Data items | 10a | List and define all outcomes for which data were sought. Specify whether all results that were compatible with each outcome domain in each study were sought (e.g. for all measures, time points, analyses), and if not, the methods used to decide which results to collect. | Lines 91-100 |
|  | 10b | List and define all other variables for which data were sought (e.g. participant and intervention characteristics, funding sources). Describe any assumptions made about any missing or unclear information. | N/A |
| Study risk of bias assessment | 11 | Specify the methods used to assess the risk of bias in the included studies, including details of the tool(s) used, how many reviewers assessed each study and whether they worked independently, and if applicable, details of automation tools used in the process. | Lines 109-123 |
| Effect measures | 12 | Specify for each outcome the effect measure(s) (e.g. risk ratio, mean difference) used in the synthesis or presentation of results. | N/A |
| Synthesis methods | 13a | Describe the processes used to decide which studies were eligible for each synthesis (e.g. tabulating the study intervention characteristics and comparing against the planned groups for each synthesis (item #5)). | Table 2 |
|  | 13b | Describe any methods required to prepare the data for presentation or synthesis, such as handling of missing summary statistics, or data conversions. | N/A |
|  | 13c | Describe any methods used to tabulate or visually display the results of individual studies and syntheses. | N/A |
|  | 13d | Describe any methods used to synthesize results and provide a rationale for the choice(s). If meta-analysis was performed, describe the model(s), method(s) to identify the presence and extent of statistical heterogeneity, and software package(s) used. | Lines 126-141 |
|  | 13e | Describe any methods used to explore possible causes of heterogeneity among study results (e.g. subgroup analysis, meta-regression). | N/A |
|  | 13f | Describe any sensitivity analyses conducted to assess the robustness of the synthesized results. | N/A |
| Reporting bias assessment | 14 | Describe any methods used to assess the risk of bias due to missing results in a synthesis (arising from reporting biases). | Lines 109-141 |
| Certainty assessment | 15 | Describe any methods used to assess certainty (or confidence) in the body of evidence for an outcome. | Lines 109-141 |
| **RESULTS** | | |  |
| Study selection | 16a | Describe the results of the search and selection process, from the number of records identified in the search to the number of studies included in the review, ideally using a flow diagram. | Fig.1 |
|  | 16b | Cite studies that might appear to meet the inclusion criteria, but which were excluded, and explain why they were excluded. | N/A |
| Study characteristics | 17 | Cite each included study and present its characteristics. | Table 2 |
| Risk of bias in studies | 18 | Present assessments of risk of bias for each included study. | Table 3 |
| Results of individual studies | 19 | For all outcomes, present, for each study: (a) summary statistics for each group (where appropriate) and (b) an effect estimate and its precision (e.g. confidence/credible interval), ideally using structured tables or plots. | Table 2 |
| Results of syntheses | 20a | For each synthesis, briefly summarise the characteristics and risk of bias among contributing studies. | Lines 572-594, Tables 2 and 3 |
|  | 20b | Present results of all statistical syntheses conducted. If meta-analysis was done, present for each the summary estimate and its precision (e.g. confidence/credible interval) and measures of statistical heterogeneity. If comparing groups, describe the direction of the effect. | N/A |
|  | 20c | Present results of all investigations of possible causes of heterogeneity among study results. | N/A |
|  | 20d | Present results of all sensitivity analyses conducted to assess the robustness of the synthesized results. | N/A |
| Reporting biases | 21 | Present assessments of risk of bias due to missing results (arising from reporting biases) for each synthesis assessed. | N/A |
| Certainty of evidence | 22 | Present assessments of certainty (or confidence) in the body of evidence for each outcome assessed. | Table 3 |
| **DISCUSSION** | | |  |
| Discussion | 23a | Provide a general interpretation of the results in the context of other evidence. | Lines 572-594 |
|  | 23b | Discuss any limitations of the evidence included in the review. | Lines 603-608 |
|  | 23c | Discuss any limitations of the review processes used. | Lines 603-608 |
|  | 23d | Discuss the implications of the results for practice, policy, and future research. | Lines 595-602 |
| **OTHER INFORMATION** | | |  |
| Registration and protocol | 24a | Provide registration information for the review, including register name and registration number, or state that the review was not registered. | N/A |
|  | 24b | Indicate where the review protocol can be accessed, or state that a protocol was not prepared. | Line 622-623 |
|  | 24c | Describe and explain any amendments to information provided at registration or in the protocol. | N/A |
| Support | 25 | Describe sources of financial or non-financial support for the review, and the role of the funders or sponsors in the review. | Line 619 |
| Competing interests | 26 | Declare any competing interests of review authors. | Lines 615-616 |
| Availability of data, code and other materials | 27 | Report which of the following are publicly available and where they can be found: template data collection forms; data extracted from included studies; data used for all analyses; analytic code; any other materials used in the review. | Line 622 |

**Fig.1. Results of the PRISMA-based search paradigm** [16]

**Identification of studies via databases and registers**

Records identified from:

Databases (n = 1210)

Repositories of clinical trials (n = 52)

Records removed *before screening*:

Duplicate records removed (n = 210)

Records removed for other reasons (n = 48)

**Identification**

Records screened

(n = 1004)

Records excluded

(n = 271)

Reports sought for retrieval

(n = 823)

Reports not retrieved

(n = 68)

**Screening**

Reports assessed for eligibility

(n = 755)

Reports excluded:

Different interventions assessed (n = 108)

Unclear methodology (n = 157)

No psychiatric diagnosis (n = 350)

Other reasons (n = 97)

Sources included in the review

(n = 43)

**Included**

**Table 2. Quality criteria checklist** [74]

| **Souce identified** | **Relevance questions** | | | | **Validity questions** | | | | | | | | | |
| --- | --- | --- | --- | --- | --- | --- | --- | --- | --- | --- | --- | --- | --- | --- |
|  | **1** | **2** | **3** | **4** | **1** | **2** | **3** | **4** | **5** | **6** | **7** | **8** | **9** | **10** |
| [20] | Y | Y | Y | Y | N | Y | Y | N | Y | N | N | Y | N | Y |
| [22] | Y | Y | Y | Y | Y | Y | Y | Y | Y | Y | Y | Y | Y | Y |
| [23] | Y | Y | Y | Y | Y | Y | Y | Y | Y | Y | Y | Y | Y | Y |
| [24] | Y | Y | Y | Y | Y | Y | Y | Y | Y | Y | Y | Y | Y | Y |
| [25] | Y | Y | Y | Y | Y | Y | N/A | N | N | Y | Y | Y | N | Y |
| [26] | Y | Y | Y | Y | Y | Y | N | Y | N | Unclear | N | Y | Y | Unclear |
| [27] | Y | Y | Y | Y | Y | Y | Y | Y | Y | Y | Y | Y | Y | Y |
| [28] | Y | Y | Y | Y | Y | Y | Y | Y | Y | Y | Y | Y | Y | Y |
| [29] | Y | Y | Y | Y | Y | Y | Y | Y | Y | Y | Y | Y | Y | Y |
| [30] | Y | Y | Y | Y | Y | Y | Y | Y | Y | N | Y | Y | N | Y |
| [31] | Y | Y | Y | Y | Y | Y | Y | Y | Y | Y | Y | Y | Y | Y |
| [35] | Y | Y | Y | Y | Y | N | Y | N | Y | Y | Y | Y | Y | Y |
| [36] | Y | Y | Y | Y | Y | Y | Y | Y | Y | N | Y | N | Y | Y |
| [37] | Y | Y | Y | Y | Y | Y | Y | Y | Y | Y | Y | Y | Y | Y |
| [38] | Y | Y | Y | Y | Y | Y | Y | Y | Y | Y | Y | Y | Y | Y |
| [32] | Y | Y | Y | Y | Y | Y | Y | Y | Y | Y | Y | Y | Y | Y |
| [39] | Y | Y | Y | Y | Y | Y | Y | Y | Y | Y | Y | Y | Y | Y |
| [40] | Y | Y | Y | Y | Y | Y | Y | Y | Y | Y | Y | Y | Y | Y |
| [49] | Y | Y | Y | Y | N | Y | Y | N | Y | Y | Y | Y | Y | Y |
| [50] | Y | Y | Y | Y | Y | Y | Y | N | Y | Y | Y | Y | Y | Unclear |
| [51] | Y | Y | Y | Y | Y | Y | N | Y | Y | Y | Y | Y | Y | Y |
| [52] | Y | Y | Y | Y | Y | N | N | N | Y | Y | Y | Y | Y | Y |
| [53] | Y | Y | Y | Y | Y | Y | Y | Y | Y | Y | Y | Y | Y | Y |
| [55] | Y | Y | Y | Y | Y | N | Y | N | Unclear | Y | Y | Y | Y | Y |
| [56] | Y | Y | Y | Y | Y | N | Y | N | Unclear | Y | Y | Y | Y | Y |
| [57] | Y | Y | Y | Y | Y | N | Y | N | Unclear | Y | Y | Y | Y | Y |
| [44] | Y | Y | Y | Y | Y | Y | Y | Y | Y | Y | Y | Y | Y | Y |
| [62] | Y | Y | Y | Y | Y | Y | N | N | N | Y | Y | Y | Y | Y |
| [64] | Y | Y | Y | Y | Y | Y | Y | Y | Y | Y | Y | Y | Y | Y |
| [65] | Y | Y | Y | Y | Y | Y | Y | Y | Y | Y | Y | Y | Y | Y |
| [66] | Y | Y | Y | Y | Y | Y | Y | Y | Y | Y | Y | Y | Y | Y |
| [67] | Y | Y | Y | Y | Y | Y | Y | Y | Y | Y | Y | Y | Y | Y |
| [68] | Y | Y | Y | Y | Y | Y | Y | Y | Y | Y | Y | Y | Y | Y |
| [73] | Y | Y | Y | Y | Y | Y | Y | Y | Y | Y | Y | Y | Y | Y |
| [78] | Y | Y | Y | Y | Y | Y | Y | Y | Y | Y | Y | Y | Y | Y |
| [76] | Y | Y | Y | Y | N | Y | Y | Y | Y | Y | N | Y | Y | Y |
| [79] | Y | Y | Y | Y | Y | Y | Y | N | Y | Y | Y | Y | Y | Y |
| [80] | Y | Y | Y | Y | Y | Y | N/A | N | N | Y | Y | Y | Y | Y |
| [81] | Y | Y | Y | Y | Y | N | N/A | N | N/A | Y | Y | Y | N | Y |
| [82] | Y | Y | Y | Y | Y | Y | Y | N | N | Y | Y | Y | Y | Y |
| [85] | Y | Y | Y | Y | Y | Y | Y | Y | Y | Y | Y | Y | Y | Y |
| [86] | Y | Y | Y | Y | Y | Y | N/A | N | N | N | Y | Y | Y | Y |
| [87] | Y | Y | Y | Y | Y | Y | N/A | N | N | N | Y | Y | Y | Y |

Relevance questions refer to (1) improving outcomes through research; (2) useful outcomes for the population; (3) the level of interest in the dietetics practice; (4) the feasibility of the procedure [11]. Validity questions refer in case of clinical trials/animal studies to (1) clearly stated research questions; (2) lack of bias in the selection process; (3) comparability of the groups; (4) withdrawal procedures; (5) blinding procedure; (6) description of the interventions; (7) definition of outcomes; (8) statistical analysis adequacy; (9) conclusions are supported by results; (10) funding/sponsorship bias. In the case of reviews/meta-analysis, the validity questions are the same, with the following modifications: (2) comprehensibility of the search strategy; (3) formulation of the inclusion/exclusion criteria; (4) quality and validity assessment; (5) description of the interventions; (6) definition of the outcomes; (7) synthesis and analysis procedure are described: (8) clarity of the results [11].
